# Multi-omics analysis identifies CLDN10 as a B-cell-associated prognostic biomarker in HPV-negative head and neck squamous cell carcinoma

**DOI:** 10.3389/fcell.2026.1788462

**Published:** 2026-03-03

**Authors:** Qihua Dang, Yayun He, Runan Zhao, Hongyuan Chen, Lijuan Yin, Yanhua Lu, Xiaocheng Shi, Yiming Li, Yanhua Chen, Yisha Gao, Miaoxia He

**Affiliations:** Department of Pathology, Changhai Hospital, Naval Military Medical University, Shanghai, China

**Keywords:** B cells, bioinformatics, CLDN10, hNSC, HPV, prognosis

## Abstract

**Background:**

The prognosis of head and neck squamous cell carcinoma (HNSC) is poor, and new biomarkers are urgently needed. Claudin 10 (CLDN10) plays an important role in various tumors, but its function in HNSC remains unclear.

**Methods:**

Through bioinformatics analysis and experimental verification, the expression, prognostic value, and immunological correlation of CLDN10 in HNSC were systematically evaluated.

**Results:**

CLDN10 is downregulated in human papillomavirus negative HNSC, and its low expression is significantly associated with a decreased overall survival of patients, and it is an independent prognostic factor. Functional enrichment analysis shows that CLDN10 and its co-expressed genes are mainly enriched in B-cell-related immune pathways. Further analysis indicates that the expression level of CLDN10 is strongly correlated with the degree of B-cell infiltration in HNSC.

**Conclusion:**

The downregulation of CLDN10 is associated with poor prognosis in HNSC, especially in HPV-negative patients, and it has significant prognostic value. Its mechanism of action may involve the regulation of B-cell-mediated tumor immune response, providing potential targets for immunotherapy and prognosis assessment of HNSC.

## 1 Introduction

 Head and neck cancers (HNC) predominantly arise in the mucosal linings of the oral cavity, oropharynx, larynx, nasopharynx, and hypopharynx. Ranking as the sixth most common cancer globally, over 90% of these cases are HNSC (Mery et al., 2016). Current statistics project approximately 54,000 new cases and an estimated 11,000 deaths in 2022 (Siegel et al., 2022). Risk factors for HNSC encompass tobacco and alcohol consumption, betel nut chewing, inadequate oral hygiene, and HPV infection (Leemans et al., 2018). As sexual behaviors evolve and HPV transmission becomes more prevalent, the incidence of HPV-related HNSC is steadily increasing (Wang M. B. et al., 2015). The therapeutic responses to chemoradiotherapy differ significantly between HPV-positive and HPV-negative HNSC patients, with HPV-negative typically facing a poorer prognosis (Shui et al., 2018). The mechanisms responsible for these differences are not yet fully understood. The primary treatment modalities for HNSC include surgery, radiotherapy, and chemotherapy, either as standalone treatments or in combination. Despite the availability of diverse therapeutic options, the overall 5-year survival rate for HNSC patients remains below 50%, posing a significant threat to human health (Park et al., 2021). Apart from cetuximab monotherapy, which targets the epidermal growth factor receptor (EGFR), no other novel targeted therapies have been approved for HNSC treatment in decades. Therefore, there is an urgent need to identify superior molecular markers that can facilitate the development of precision medicine and personalized treatment strategies for HNSC. Notably, research into HPV-related HNSC is particularly significant, offering both clinical relevance and substantial social value.

Claudins (CLDNs), derived from the Latin word ‘claudere’ meaning ‘to close’, were identified by Tsukita in 1998 as the major integral membrane proteins that formed tight junction (TJ) strands (Furuse et al., 1998). CLDN proteins are 20–34 kDa in size and the structure of the various claudins is very similar and includes four transmembrane domains (TM1-4), the intracellular N and C termini, and two extracellular loops (ECL1 and ECL2) (Suzuki et al., 2014). The CLDN protein family comprises 27 members that function as skeleton proteins within tight junctions (TJs) (Singh and Dhawan, 2015). CLDNs possess a barrier function that regulates epithelial polarity and vectorial movement of solutes and fluids in the intercellular space (Tsukita et al., 2019). For instance, the deletion of CLDN1 compromises the epidermal barrier, leading to rapid lethality within 1 day due to excessive water loss (Furuse et al., 2002). Loss of CLDN7 resulted in the aberrant regulation of small organic solute paracellular flux across the colon epithelium, leading to colonic inflammation (Tanaka et al., 2015). Additionally, CLDNs have a fence function that controls the lateral diffusion of proteins within the lipid bilayer (Otani and Furuse, 2020). Apart from the obvious role of creating scaffolds, CLDNs are involved in signal transduction within the cell. For example, a positive feedback regulation between CLDN1 and Wnt-signaling, the key regulator of colon carcinogenesis (Dhawan et al., 2005). CLDN6 is found to be upregulated and interacts with ZO-2/YAP1, activating the Hippo signaling pathway in hepatocellular carcinoma (HCC) cells (Kong et al., 2021). Studies have shown that dysregulation of CLDN-mediated barrier function and signaling is a precursor to cancer pathogenesis (Zihni et al., 2016).

CLDN10, a crucial member of this protein family, is widely expressed in various organs and tissues. In 2017, a study confirmed that mutations in the CLDN10 gene could lead to hypohidrosis and electrolyte imbalance (HELIX) (Bongers et al., 2017), which garnered significant attention. Apart from its involvement in kidney function, CLDN10 has been implicated in various tumor developments. For instance, it promotes the malignant phenotype of osteosarcoma cells through JAK1/Stat1 signaling (Zhang et al., 2019). Additionally, it enhances the metastatic ability of melanoma cells via activation of the ERK pathway (Perez et al., 2017). Furthermore, CLDN10 serves as a novel biomarker for predicting prognosis in patients with ovarian cancer (Li Z. et al., 2020) and metastatic high-grade serous carcinoma (Davidson et al., 2023). Overexpression of CLDN10 suppresses growth and metastasis in human clear cell renal cell carcinoma (Yang et al., 2022), whereas its upregulation is associated with overall survival (OS) in hepatocellular carcinoma patients (Huang et al., 2011). Although several studies have examined the interaction, regulatory mechanisms, and localized expression of claudin-10 with other closely associated molecules, the precise role and mechanisms of CLDN10 in various tumors, particularly HNSC, remain to be fully elucidated.

Therefore, the aim of this study is to utilize a range of bioinformatics approaches, in conjunction with *in vitro* experiments, to conduct a preliminary investigation into the expression of CLDN10 in HNSC and to explore its potential mechanisms.

## 2 Materials and methods

### 2.1 Datasets and patients

We obtained HNSC RNA-seq datasets, along with corresponding phenotypic, survival, and clinical information, from The Cancer Genome Atlas (TCGA) database (Liu et al., 2018) via the UCSC XENA online platform (https://xena.ucsc.edu/). The raw data were imported into R software (version 4.3.2) and visualized using tidy-verse and ggpubr packages. The Wilcoxon rank sum test was used to compare two datasets, with *p* < 0.05 considered statistically significant. Significance levels were indicated as ns (not significant, *p* ≥ 0.05), * (*p* < 0.05), ** (*p* < 0.01), *** (*p* < 0.001, and **** (*p* < 0.0001).

We collected 112 primary HNSC tumor tissue samples, along with their paired peritumoral or normal tissues, from Changhai Hospital, Naval Military Medical University between 1 January 2017, and 31 December 2023. This cohort included 55 cases of oropharyngeal carcinoma, 21 cases of nasopharyngeal carcinoma, 27 cases of laryngeal carcinoma, and 9 cases of hypopharyngeal carcinoma. Formalin-fixed paraffin-embedded (FFPE) tissues were sectioned to a thickness of 4 μm to obtain sequential sections for immunohistochemical staining and analysis. All patients obtained informed consent.

### 2.2 Differential expression analysis

GEPIA (http://gepia.cancer-pku.cn/index.html) database analyzed CLDNs mRNA expression in HNSC compared to normal head and neck tissues. Kaplan-Meier plotter (http://kmplot.com/analysis/) database was used to analyze the relationship between CLDNs expression level and OS rate among HNSC patients. The UALCAN (https://ualcan.path.uab.edu/index.html) online website analyzed the protein expression of CLDN10 in HNSC within the CPTAC database. Based on the immunohistochemical results of 12 normal head and neck tissues and 4 HNSC samples from the Human Protein Atlas (HPA) (https://www.proteinatlas.org) (antibody: CAB012969), the protein expression differences of CLDN10 were evaluated. In terms of spatial localization, the specific expression location of CLDN10 in salivary gland tissues was clarified through a multiplex immunofluorescence experiment (antibody: HPA042348).

### 2.3 Overall survival and prognosis analysis

The HNSC RNA-seq data, phenotypic and survival data from TCGA were imported into RStudio. Firstly, a Log-rank test was performed to analyze the relationship between CLDN10 expression in HNSC and OS, Disease Specific Survival (DSS) and Progress Free Interval (PFI). Subsequently, 32 HPV-positive and 80 HPV-negative samples were selected for further analysis. Perform the proportional hazards test using the survival package and conduct the Cox regression analysis. The association between CLDN10 expression and OS in HNSC, as well as within the HPV-positive and HPV-negative cohorts, was assessed using the Wilcoxon rank sum test to compare the two groups. Visualization of the results was done using packages such as survival, survminer, and ggpubr.

### 2.4 Gene set enrichment analysis

The Linked-Omics (https://www.linkedomics.org/) database identified co-expressed transcripts with CLDN10 in 517 patients (520 microarrays) from the TCGA-HNSC cohort [ *p* < 0.01, false discovery rate (FDR) < 0.05]. To assess their prognostic significance in HNSC, heatmaps visualizing the top 30 positively correlated transcripts along with negatively correlated ones were generated using GEPIA2. Enrichment analyses utilized Link-Interpreter module where ‘Overrepresentation Analysis’ ranked positively correlated transcripts based on p-value criteria while ‘Gene Set Enrichment Analysis (GSEA)' employed p-value ranking with 500 simulations performed. Enriched terms obtained from these analyses underwent further processing using ‘Affinity propagation’ method to reduce redundancy. Clusters showing a p-value <0.05 were considered statistically significant.

### 2.5 Immune cell infiltration analysis

We analyzed the correlation between CLDN10 expression and infiltration of immune cells in HNSC using the TIMER2 database (https://cistrome.shinyapps.io/timer/). The immune cells included B cells, CD4^+^ T cells, CD8^+^ T cells, neutrophils, macrophages, and dendritic cells. Spearman’s correlation coefficient between gene expression and immune cell infiltration scores in HNSC was calculated using the corr function from the R package psych (version 4.3.2). The correlation between CLDN10 and CD20, CD38, CD8, CD4, CD68 and CD163 in HNSC was analyzed by GEPIA. Spearman’s rank correlation tests were conducted to assess these associations, with a p-value of less than 0.05 considered statistically significant.

### 2.6 Immunohistochemistry (IHC) analysis

Hematoxylin and eosin (H&E) staining was performed on each tissue sample. A standard protocol was followed to control morphological quality. The expression of CLDN10, CD4, CD8, CD20, CD38, CD68 and CD163 in HNSC was detected by automatic immunohistochemical staining. Paraffin sections of human HNSC were dewaxed using xylene and graded alcohols. Then, the sections were immersed in EDTA buffer (pH = 9.0) (95 °C–98 °C, 5 min) for antigen retrieval. The sections were treated with 3%–4% hydrogen peroxide (H_2_O_2_) block for 5 min to inactivate endogenous peroxidase activity. Then, the sections were incubated with rabbit anti-human CLDN10 (1:400 dilution, Abcam, United States) for 15 min. Antibodies for CD4, CD8, CD20, CD38, CD68, and CD163 were applied at their ready-to-use, undiluted concentrations. Sections were then incubated with biotinylated goat anti-rabbit secondary antibody for 10 min. Diaminobenzidine (DAB) was used for color development. Positive controls used thyroid cancer tissue, which was treated in the same way. CLDN10 was expressed in cell membrane/cytoplasm, and yellow or brown-yellow staining in cell membrane/cytoplasm was positive. The staining was graded for intensity (0-negative, 1-weak, 2-moderate, and 3-strong) and percentage of positive cells as follows: 0–1, <5% positive tumor cells; 2, 5%–20% positive tumor cells; 3, 20%–40% positive tumor cells; 4, 40%–60% positive tumor cells; 5, >60% positive tumor cells (Allred et al., 1993). The cell counting was repeated in five randomly-selected microscopic fields at ×400 magnification. The total score of each section: 0–5, low expression; five to eight, high expression.

### 2.7 HPV-tumor status

HPV-tumor status of the HNSC was assessed using a combination of p16 IHC staining (p16, a surrogate for HPV + HNSC) and HPV-DNA ISH test. Only patients with positive for both p16 IHC and ISH test were assigned a positive HPV tumor status. Paraffin sections of human HNSC were dewaxed using xylene and graded alcohol. Then, the sections were immersed in boiling distilled water for 5 min for antigen retrieval. Then 70–100 μL pepsin was added onto slides to completely cover the sample tissue. The slides were incubated at 37 °C for 5 min. Then 70–100 μL HPV probe was added to the tissue area. The slides were incubated at 37 °C for 120 min. Then 70–100 μL signal amplification probe I, signal amplification probe II, and signal amplification probe III were added the slides, respectively. The slides were incubated at 37 °C. After 70–100 μL of digoxin antibody was added onto the tissue, the slides were placed in the wet box with a constant temperature and humidity and were incubated at 37 °C for 30 min. Finally, diaminobenzidine (DAB) and hematoxylin color development were performed. The presence of spotty positive staining in the nucleus indicates that HPV virus DNA is integrated into the genomic DNA of human cells. The presence of diffuse positive staining in the cytoplasm indicates that HPV-RNA has begun to express.

### 2.8 Statistical analysis

All data were analyzed using SPSS software (version 27). The Nonparametric rank sum test (Mann-Whitney U) was used to analyze the relationship between the clinical features of HNSC and the expression of CLDN10. The agreement of expression between CLDN10 and markers CD4, CD8, CD20, CD38, CD68, and CD163 in HNSC was assessed using the Kappa assay. Kappa ≥0.85, indicating good consistency of test results. 0.6 ≤ Kappa <0.85, it is considered that the consistency of test results is good; 0.45 ≤ Kappa <0.6, the consistency of test results was considered to be general; Kappa <0.45, indicating poor consistency of test results. *p* < 0.05 is considered statistically significant.

## 3 Results

### 3.1 Differential expression of CLDNs in HNSC

**FIGURE 1:**
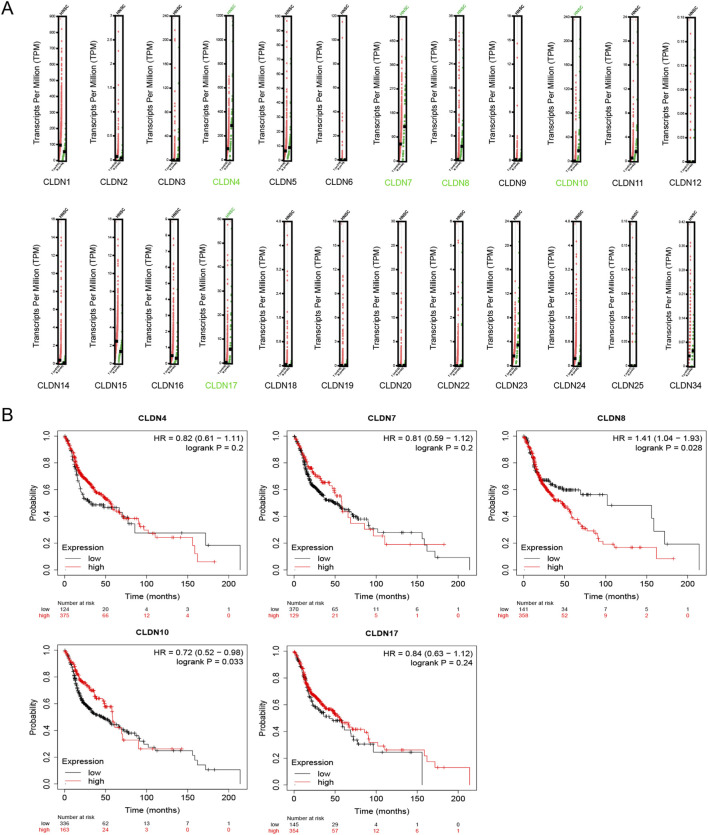
Differential expression of CLDNs in HNSC and its relationship with OS based on TCGA samples. **(A)** The mRNA expression of CLDNs in HNSC. *p* < 0.01. **(B)** The prognostic value of CLDNs in HNSC patients in the OS curve.

GEPIA database analysis revealed that CLDN4, CLDN7, CLDN8, CLDN10, and CLDN17 were significantly downregulated in HNSC compared to normal head and neck tissues (Figure 1A). Kaplan-Meier plotter survival analysis revealed that only the low expression of CLDN10 was significantly associated with a shortened overall survival period for the patients, suggesting that it may have important prognostic value in HNSC (Figure 1B). Therefore, subsequent studies will focus on analyzing the expression and role of CLDN10 in HNSC.

### 3.2 Downregulation of CLDN10 expression in HNSC

**FIGURE 2:**
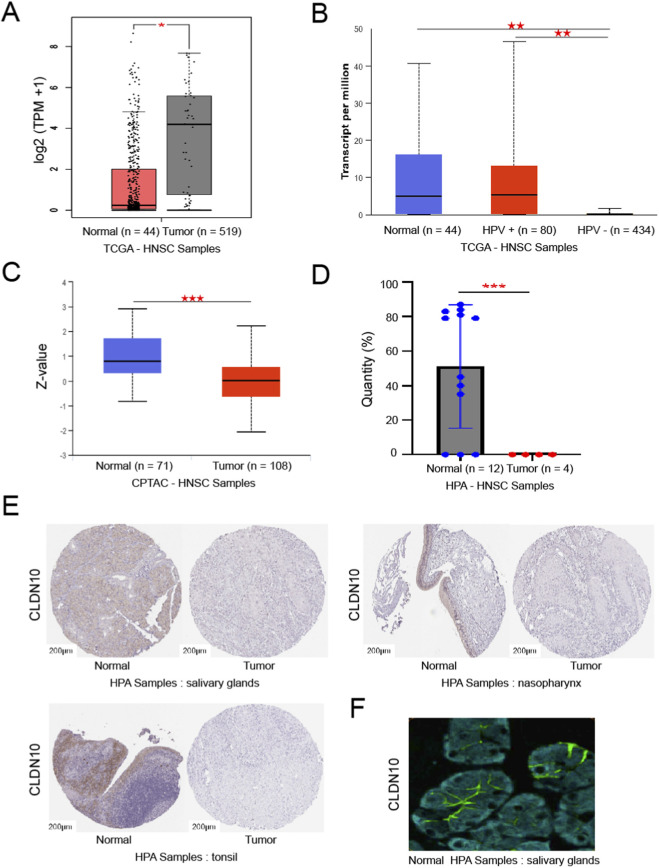
The CLDN10 expression levels in HNSC patients. *p < 0.05, **p < 0.01, ***p < 0.001. **(A)** The mRNA expression of CLDN10 in HNSC based on TCGA samples. **(B)** The expression of CLDN10 mRNA in HNSC based on the HPV infection status of patients in the TCGA samples. **(C)** The protein expression of CLDN10 in HNSC based on CPTAC samples. **(D)** The protein expression of CLDN10 in HNSC based on HPA database. **(E)** Representative images of immunohistochemical staining in the HPA database. **(F)** Expression and localization of CLDN10 protein in cells based on HPA database.

Analysis of the GEPIA and UALCAN databases revealed that in HNSC, the mRNA expression level of CLDN10 was significantly lower than that in normal tissues (Figure 2A); moreover, the expression level in HPV-negative patients was significantly lower than that in HPV-positive patients (Figure 2B). Based on the high-throughput proteomics technology analysis of HNSC in the CPTAC database, the expression level of CLDN10 protein in tumor tissues was significantly lower than that in normal tissues (Figure 2C). Based on the analysis of the HPA database, the expression of the CLDN10 protein in HNSC was significantly lower than that in normal head and neck tissues (Figure 2D). This trend was also confirmed in comparisons of various normal tissues such as salivary glands, nasopharynx, and tonsils (Figure 2E). Further multiplex immunofluorescence experiments demonstrated that CLDN10 was mainly located on the cell membrane in salivary gland tissues (Figure 2F). These results collectively indicate that CLDN10 is expressed at a lower level in HNSC, especially in HPV-negative subtypes.

### 3.3 High CLDN10 expression levels associated with better survival rates in HNSC patients

**FIGURE 3:**
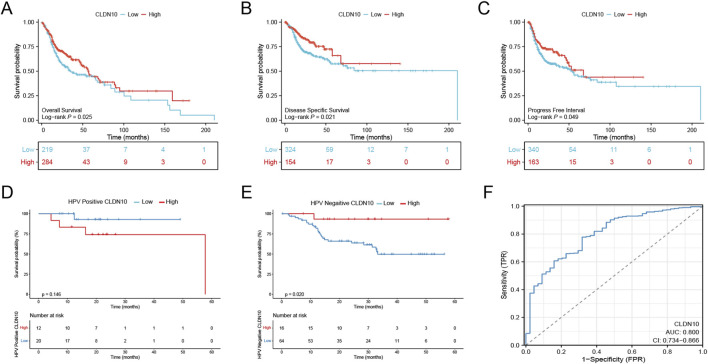
The survival analysis of CLDN10 mRNA levels in HNSC patients based on TCGA samples. **(A–C)** The OS, DSS and PFI survival rates in patients with high and low CLDN10 levels. **(D,E)** The prognostic value of CLDN10 in HNSC patients, HPV-positive-HNSC and HPV-negative-HNSC patients in the OS curve. **(F)** ROC curve analysis for CLDN10 expression in HNSC.

Survival analysis indicated that high expression of CLDN10 was significantly associated with longer OS, DSS, and PFI in HNSC patients (Figures 3A–C), and this association was more pronounced in HPV-negative patients (Figures 3D,E). Receiver Operating Characteristic (ROC) curve analysis revealed that CLDN10 had good diagnostic discriminatory ability for HNSC (AUC = 0.800, Figure 3F), suggesting its potential as a prognostic and diagnostic biomarker. However, its clinical transformation still needs further verification. Univariate analysis of correlation revealed that some factors, including T stage, N stage,M stage, and CLDN10 expression are significantly correlated with OS. Our multivariate analysis, revealed that CLDN10 expression is an independent factor for prognosis, suggesting that the higher the expression of CLDN10, the better the prognosis of the patients (Table 1).

**TABLE 1:** Correlation between OS and multivariable characteristics in TCGA patients via Cox regression analysis.

Characteristics	Total	Univariate analysis		Multivariate analysis	
		Hazard radio (95% CI)	P value	Hazard radio (95% CI)	P value
T stage	447	​	​	​	​
T1	45	Reference	​	Reference	​
T2	134	1.341 (0.694–2.590)	0.382	0.717 (0.084–6.116)	0.761
T3	96	2.592 (1.349–4.984)	0.004	1.286 (0.155–10.645)	0.816
T4	172	2.347 (1.250–4.407)	0.008	1.847 (0.230–14.850)	0.564
N stage	410	​	​	​	​
N0	170	Reference	​	Reference	​
N1	66	0.957 (0.563–1.624)	0.87	0.826 (0.299–2.282)	0.712
N2&N3	174	2.269 (1.617–3.183)	<0.001	3.328 (1.615–6.859)	0.001
M stage	188	​	​	​	​
M0	187	Reference	​	Reference	​
M1	1	22.631 (2.830–180.948)	0.003	35.560 (2.668–473.946)	0.007
Gender	503	​	​	​	​
Female	134	Reference	​	Reference	​
Male	369	0.760 (0.571–1.012)	0.061	0.648 (0.356–1.179)	0.156
Age	503	​	​	​	​
≤ 60	247	Reference	​	Reference	​
>60	256	1.262 (0.964–1.653)	0.09	0.906 (0.514–1.598)	0.734
Smoker	493	​	​	​	​
No	113	Reference	​	​	​
Yes	380	1.110 (0.793–1.554)	0.544	​	​
Alcohol history	492	​	​	​	​
No	159	Reference	​	​	​
Yes	333	0.954 (0.718–1.268)	0.745	​	​
CLDN10	503	0.820 (0.701–0.960)	0.013	0.780 (0.638–0.953)	**0.015**

*P* < 0.05: This is referred to as “statistically significant”.

### 3.4 CLDN10 gene set enrichment analysis in HNSC

**FIGURE 4:**
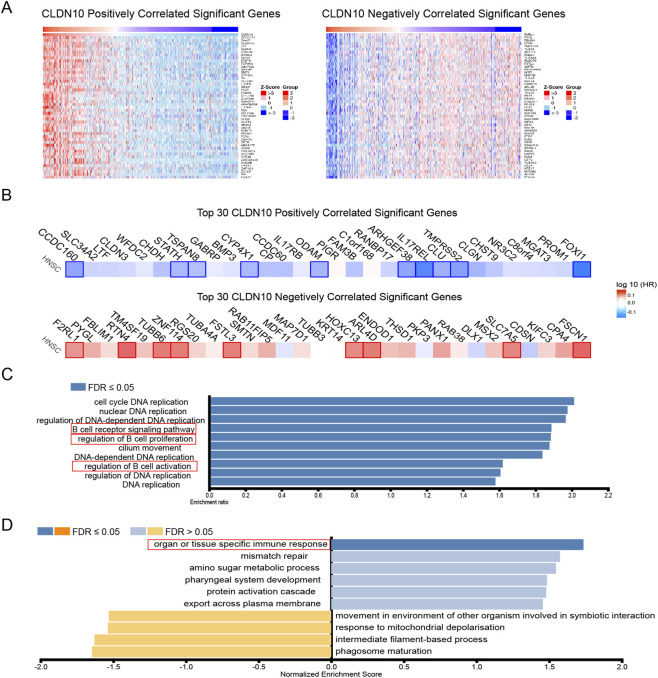
CLDN10 Gene set enrichment analysis in HNSC based on TCGA samples. **(A)** The top 50 positively and negatively correlated transcripts. **(B)** The top 30 positively and negatively correlated transcripts in the overall survival of HNSC patients (blue color, negative prognostic predictor; red color, positive prognostic predictor). **(C)** The bar diagram shows the co-expressed transcripts with significant enrichment in GO annotations for Biological Process. **(D)** The bar diagram shows the co-expressed transcripts with significant enrichment in GSEA identifies enriched KEGG pathways.

Figure 4A illustrates the top 50 genes positively associated with CLDN10 and the top 50 genes negatively associated with it. Among the top 30 altered transcript, 10 positively and 9 negatively correlated transcripts were significantly predictive of the overall survival of HNSC (Figure 4B), indicating a strong impact of the CLDN10 network on the pathogenesis of HNSC. Given their strong associations with CLDN10 levels, these aforementioned nineteen transcripts potentially contribute to survival differences through co-regulation mechanisms involving CLDN10 levels. We performed GO enrichment analysis using Linked-Omics website on co-expressed transcripts (Figure 4C) revealed that biological processes primarily related to “DNA replication” and “B cell receptor signaling pathway”,”B cell proliferation” and “B cell activation” were highly enriched among them. GSEA demonstrated enriched KEGG pathways (Figure 4D), predominantly focusing on “organ or tissue specific immune response”. Bioinformatics analysis revealed that the expression pattern of CLDN10 was significantly enriched in the B-cell-mediated immune pathways, suggesting a potential association between CLDN10 and B-cell function in the tumor immune microenvironment of HNSC. Moreover, the decreased expression level of CLDN10 was consistent with the weakened B-cell-related immune response in the tumor microenvironment, and both were associated with the pathological features of tumor immune escape.

### 3.5 The relationship between the CLDN10 level and tumor immune infiltration

**FIGURE 5:**
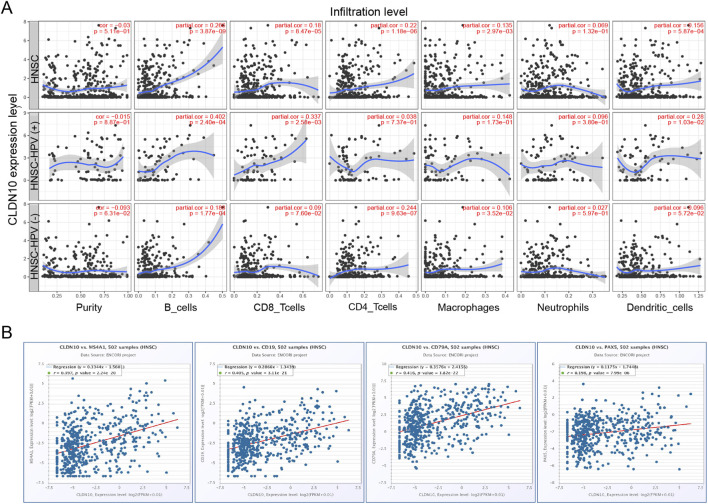
The relationship between the CLDN10 level and tumor immune infiltration. **(A)** Association of CLDN10 levels with immune cell infiltrates in HNSC based on TIMER2 database. **(B)** Association of CLDN10 levels with B cell related markers in HNSC.

Enrichment analysis results suggest that CLDN10 is likely an immune-related factor in HNSC. We used the TIMER2 database to analyze the correlation between CLDN10 expression and six immune cell types in HNSC. As shown in Figure 5A, there was a significant positive correlation between CLDN10 expression and B cells, CD8^+^ T cells, CD4^+^ T cells, macrophages, and dendritic cells in HNSC, among which the correlation with B cells was the highest. We further analyzed the correlation between CLDN10 expression and B immune cell-related markers in HNSC: CD20, CD19, CD79A, and PAX5. The results showed that the expression level of CLDN10 was highly correlated with B cell (Figure 5B).

### 3.6 Expression levels of CLDN10 in HNSC tissues and adjacent non-neoplastic tissues

**FIGURE 6:**
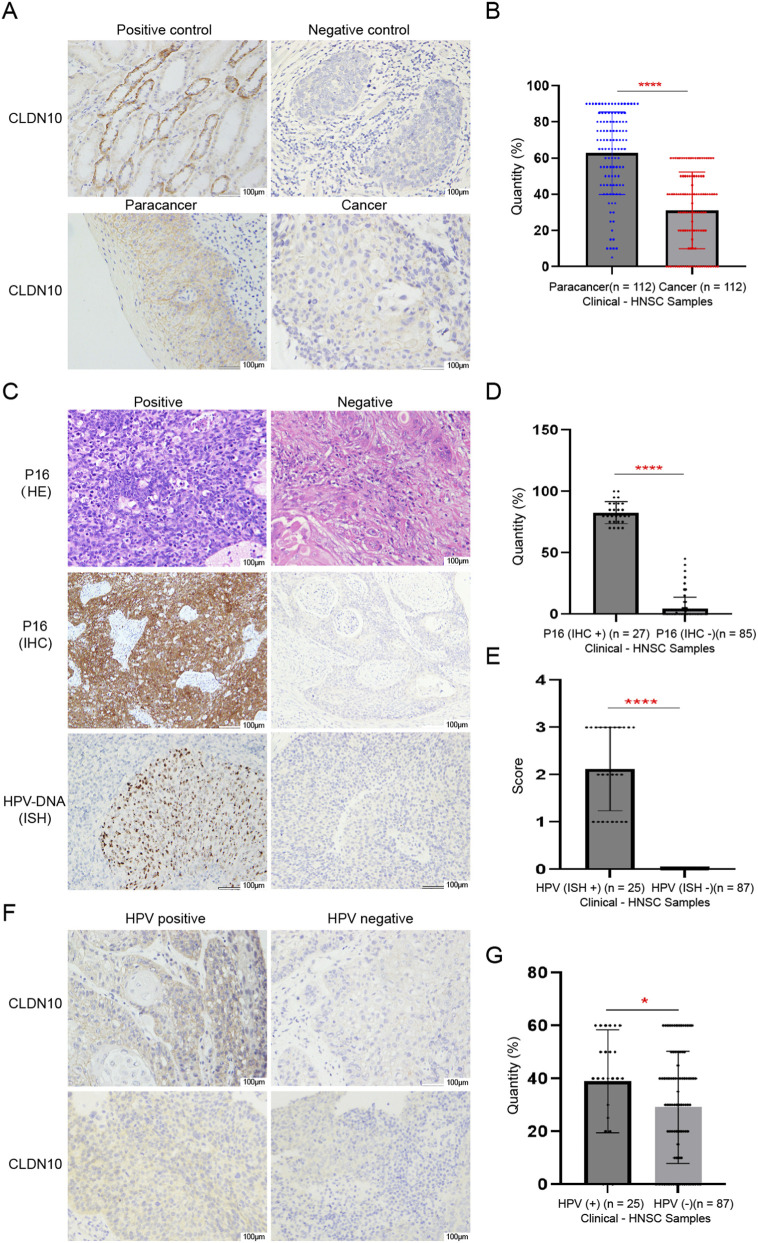
The CLDN10 expression levels in HNSC patients based on clinical samples. *p < 0.05, **p < 0.01, ***p < 0.001,****p < 0.0001. **(A)** Representative image of CLDN10 immunohistochemical staining in HNSC tissues and adjacent non-neoplastic tissues. **(B)** Quantitative analysis of the expression level of CLDN10 in each group shown in [A] based on the positive expression rate of IHC. **(C)** Representative images of HE staining, IHC and HPV-DNA ISH of P16. Among them, the HE staining under the microscope of P16 positive is mostly non-keratinizing squamous cell carcinoma, with a high nuclear-cytoplasmic ratio of tumor cells and frequent mitotic figures; the HE staining under the microscope of P16 negative is mostly moderately to well-differentiated squamous cell carcinoma, with visible cell keratinization. **(D)** Quantitative analysis of the expression level of P16 in each group shown in [C] based on the positive expression rate of IHC. **(E)** Quantitative analysis of the expression level of HPV in each group shown in [C] based on the ISH score. **(F)** Representative image of CLDN10 immunohistochemical staining in HPV-positive-HNSC tissues and HPV-negative-HNSC tissues. **(G)** Quantitative analysis of the expression level of CLDN10 in each group shown in **(F)** based on the positive expression rate of IHC.

To further verify CLDN10 expression in HNSC, we collected 112 clinical samples to analyze protein levels in cancer and adjacent tissues. The results show that CLDN10 expression in HNSC occurs mainly in the cell membranes and is significantly decreased in cancer tissues compared to adjacent normal tissues (Figures 6A,B). Moreover, we divided 112 clinical samples into 25 HPV-positive groups and 87 HPV-negative groups according to the results of P16 IHC and HPV-DNA ISH test (Figures 6C–E). Results showed that CLDN10 expression was significantly reduced in the HPV-negative groups compared to the HPV-positive groups (Figures 6F,G).

### 3.7 Relationship of expression CLDN10 with clinicopathological features in HNSC patients

To investigate the correlation between CLDN10 expression and clinical characteristics in HNSC patients, we examined its levels across various clinical specimen categories. We divided the cohort into two subgroups (CLDN10-low, n = 87; CLDN10-high, n = 25) base on immunohistochemical staining scores, with 0–4 defined as low expression and 5–8 as high expression. The expression level of CLDN10 showed significant correlations with Clinical T stage (P = 0.034),OS event (P = 0.011) and HPV status (P = 0.021) (Table 2). The findings suggest that CLDN10 plays a pivotal role in the progression of HNSC, and it can serve as a valuable prognostic biomarker for HNSC.

**TABLE 2:** Correlation between CLDN10 expression and the clinicopathological features of the HNSC cases from clinical specimens.

Characteristics	Low expression of CLDN10	High expression of CLDN10	P value
n	83	29	​
Clinical T stage, n (%)	​	​	0.034
T1	33 (29.5%)	8 (7.1%)	​
T2	26 (23.2%)	5 (4.5%)	​
T3	13 (11.5%)	12 (14.8%)	​
T4	11 (9.8%)	4 (3.6%)	​
Clinical N stage, n (%)	​	​	0.367
N0	38 (33.9%)	9 (8.0%)	​
N1	17 (15.2%)	7 (6.3%)	​
N2	25 (22.3%)	10 (9.0%)	​
N3	3 (2.7%)	3 (2.7%)	​
Clinical M stage, n (%)	​	​	0.554
M0	82 (73.2%)	29 (25.9%)	​
M1	1 (0.9%)	0 (0%)	​
Gender, n (%)	​	​	0.810
Female	16 (14.3%)	5 (5.5%)	​
Male	67 (59.8%)	24 (21.4%)	​
Age, n (%)	​	​	0.969
≤ 60	34 (30.4%)	12 (10.6%)	​
>60	49 (43.8%)	17 (15.2%)	​
Smoker, n (%)	​	​	0.540
No	34 (30.4%)	10 (8.9%)	​
Yes	49 (43.8%)	19 (16.9%)	​
OS event, n (%)	​	​	0.011
Alive	74 (66.1%)	20 (17.9%)	​
Dead	9 (8.0%)	9 (8.0%)	​
HPV, n (%)	​	​	0.021
Negative	60 (53.6%)	27 (24.1%)	​
Positive	23 (20.5%)	2 (1.8%)	​

### 3.8 The relationship between the CLDN10 level and immune cell related markers in HNSC patients

**FIGURE 7:**
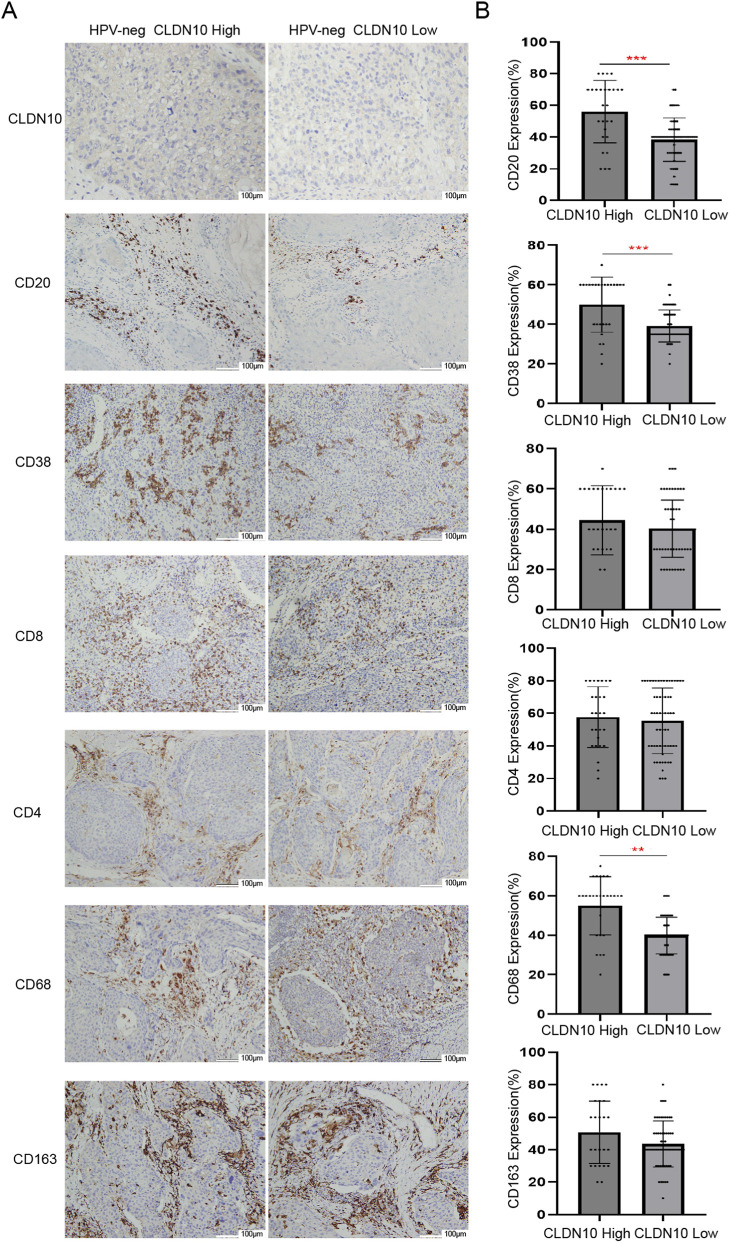
Expression levels of CLDN10 and immune cell-related markers in HPV-negative HNSC patients based on clinical samples. ** p < 0.01, *** p < 0.001. **(A)** Representative images of immunohistochemical staining of immune cell-related markers in the CLDN10-high and low group. **(B)** Based on the positive expression rate of IHC, quantitative analysis of the expression levels of immune cell-related markers in [A].

Based on the analysis of the TCGA-HNSC cohort, we observed a strong correlation between CLDN10 expression and B cells. To validate this finding, we further categorized 87 HPV-negative-HNSC patients into CLDN10-low groups and CLDN10-high groups based on immunohistochemical staining scores. Subsequently, we assessed the levels of six immune cell-related markers using IHC in each group. The results revealed that in the HPV-negative-HNSC groups, the CLDN10-low groups showed a significant reduction in CD20 and CD38 levels compared to the CLDN10-high groups while no notable differences were observed in CD8, CD4, CD68 or CD163 levels (Figures 7A,B). Additionally, the Kappa consistency test showed poor agreement between CLDN10 expression and six immune cell-related markers in HPV-negative HNSC groups. However, good consistency was observed between CLDN10 and CD20 expression (Kappa >0.6), indicating agreement (Table 3). A P value <0.05 was considered statistically significant.

**TABLE 3:** Correlation between CLDN10 expression and immune cell related markers of the HPV-negative-HNSC groups from clinical specimens.

Marks	Low expression of CLDN10	High expression of CLDN10	Kappa	P value
n	60	27	​	​
CD20	​	​	0.668	<0.01
Low expression	51	4	​	​
High expression	9	23	​	​
CD38	​	​	0.409	<0.01
Low expression	49	11	​	​
High expression	11	16	​	​
CD4	​	​	0.067	0.433
Low expression	23	8	​	​
High expression	37	19	​	​
CD8	​	​	0.140	0.190
Low expression	42	15	​	​
High expression	18	12	​	​
CD68	​	​	0.494	<0.01
Low expression	47	7	​	​
High expression	13	20	​	​
CD163	​	​	0.155	0.134
Low expression	37	12	​	​
High expression	23	15	​	​

## 4 Discussion

HNSC is characterized by a complex anatomical structure and diverse tumor types that often have an insidious onset. The malignancy of HNSC is such that up to 30% of patients experience cancer relapse and treatment failure (Canning et al., 2019). While tumor immunotherapy has recently shown promising results, it is not universally effective due to individual differences and tumor heterogeneity (Ferris et al., 2018). Therefore, it is crucial to analyze prognostic factors and elucidate the molecular mechanisms of HNSC. Concurrently, there is a critical need to search for molecular markers that can facilitate precision medicine approaches, specifically tailored for personalized treatment strategies for HNSC.

A large body of evidence highlights that CLDNs play a role in nearly all aspects of tumor biology and all steps of tumor development, including inflammation, growth, survival, proliferation, epithelial-mesenchymal transition (EMT), metastasis, therapy resistance and cancer stem cell (CSC) renewal (Gowrikumar et al., 2019). For example, CLDN5 can be used to distinguish mesothelioma from angiosarcoma (Nakashima et al., 2020). The expression of CLDN6 is upregulated in hepatocellular carcinoma and can be used as a therapeutic target for HCC lineage plasticity (Kong et al., 2021; Lu et al., 2021). In gastric cancer, high CLDN7 expression correlates with shorter OS, while high CLDN18 expression correlates with longer OS (Yang et al., 2018). Moreover, numerous studies have demonstrated aberrant expression patterns of CLDNs in malignant tumors originating from various epithelial tissues,and they can serve as prognostic markers in lung cancer (Wang Q. et al., 2015), prostate cancer (Kind et al., 2020), cervical cancer (Akimoto et al., 2018), ovarian cancer (Martin de la Fuente et al., 2018), breast cancer (Jaaskelainen et al., 2018), endometrial cancer (Micke et al., 2014), and Gastric Cancer (Hashimoto and Oshima, 2022). These results suggest that claudins can be used as a biomarker to determine diagnosis, prognosis, and treatment. In conclusion, various members of the CLDN family, including CLDN10, have been documented to play a pivotal role in the genesis and progression of multiple malignancies. Currently, targeted therapies against CLDN18.2 have seen successful commercialization, offering hope to patients with advanced gastric cancer. Additionally, chimeric antigen receptor T (CAR-T) cell therapy directed at CLDN6 in solid tumors has achieved new milestones. As a significant member of the CLDN family, we speculate whether CLDN10 can be the next new therapeutic target for malignant tumors. Few relevant studies exist on the role of CLDN10 in malignant tumors, and research on CLDN10 in HNSC remains lacking; therefore, we conducted this study.

The immunohistochemical staining results showed that compared with normal head and neck tissues, the level of CLDN10 in HNSC tissues was significantly decreased, and the expression level was even lower in the HPV-negative subgroup. Survival analysis indicated that low expression of CLDN10 was significantly associated with shortened OS in HNSC patients, and was an independent prognostic factor in the multivariate analysis. Moreover, compared with HPV-positive patients, HPV-negative patients had a worse prognosis. Studies report that HPV-positive patients with tongue squamous cell carcinoma exhibit increased sensitivity to radiotherapy and chemotherapy, which leads to better prognosis (Gillison et al., 2008). Similarly, patients with HPV-associated oropharyngeal squamous cell carcinoma generally have a favorable prognosis (Massano et al., 2006). These reports are consistent with our research. Further clinical correlation analysis indicated that the expression level of CLDN10 was significantly correlated with clinical T stage, OS events, and HPV status. That is, patients with a heavier tumor burden and poorer prognosis showed lower CLDN10 expression. The receiver operating characteristic (ROC) curve analysis revealed that CLDN10 had good diagnostic discriminatory ability for HNSC (AUC = 0.800), suggesting its potential value as a prognostic and diagnostic biomarker. However, its clinical translation still requires clarification of the diagnostic threshold through prospective cohort studies and its evaluation together with existing markers (such as PD-L1, HPV status) to determine its practical value in the early screening or stratified management of HNSC. The expression of CLDNs in epithelial cells is a dynamic equilibrium pattern (Gunzel and Yu, 2013). Based on these findings, we hypothesize that the progression of HNSC may be accompanied by the downregulation of CLDN10. This change may disrupt the structural integrity and barrier function of tight junctions, thereby promoting local invasion and progression of the tumor. This is consistent with the previous research reports that the expression of Claudin family members is dysregulated in various epithelial-derived malignant tumors and affects prognosis. These findings imply that CLDN10 may act as an inhibitor of HNSC progression and could potentially serve as an independent prognostic marker for HNSC patients. Hence, it is essential to further clarify the role of CLDN10 in the pathogenesis of HNSC.

To explore the potential biological functions of CLDN10 in HNSC, we conducted GO and GSEA enrichment analyses. The results showed that the expression of CLDN10 was significantly correlated with B-cell-related immune pathways The rationale behind the metastatic and recurrence of HNSC is likely due to the interactions of the surrounding tissue matrix and immune cells that make up the tumor microenvironment (TME) (Chen et al., 2020). As a subgroup of cells in TME, B cells play an increasingly important role in tumor immunity. B cells have been shown to contribute to tumor immune escape in a variety of TME, including HNSC (Jeske et al., 2020), BRCA (Ragonnaud et al., 2019), Lung cancer (Tousif et al., 2021) and stomach cancer (Murakami et al., 2019). It has been found that B cells play an immune escape role through classical intercellular contact and secretion of cytokines, immunoglobulins, and small molecule metabolites (Li et al., 2022). Regulatory B cells (Breg) secretes TGF-β1, mediating immune escape from colorectal cancer (Wang et al., 2022). Immunoglobulin A (IgA) can mediate tumor immune escape (Zhong et al., 2021). In addition, it has been found that IgG has a pro-tumor effect in hepatocellular carcinoma (Wu et al., 2023). Adenosine (ADO) has been found in tumor tissue and peripheral blood of HNSC patients, and it is speculated that ADO binds to regulatory Breg to inhibit BCR signaling pathway (Lechner et al., 2019). Based on this, we further evaluated the correlation between CLDN10 expression and the level of immune cell infiltration in the tumor microenvironment of HNSC. We found that CLDN10 was only significantly positively correlated with the abundance of B cell infiltration. To verify this finding, we conducted immunohistochemical staining in an independent HPV-negative HNSC cohort. The results showed that the infiltration density of CD20^+^ B cells and CD38^+^ plasma cells in the CLDN10 high-expression group was significantly higher than that in the CLDN10 low-expression group. These multiple pieces of evidence indicate that there is a stable and reproducible association between CLDN10 and the infiltration of B cells in HPV-negative HNSC. However, it is important to note that the current research conclusions are limited to the level of correlation. Tumor-infiltrating B cells form a highly heterogeneous group, and their functions exhibit significant duality: they include effector B cells and plasma cells that exert anti-tumor effects through antigen presentation, antibody secretion, and germinal center reactions, as well as regulatory B cells that mediate immunosuppression by secreting inhibitory cytokines such as IL-10 and TGF-β. This study is unable to precisely distinguish the functionally distinct B cell subpopulations based on the available data. Therefore, the increase in B cell infiltration associated with high CLDN10 expression may represent an active anti-tumor immune response, or it may reflect the enrichment of immunosuppressive B cell subpopulations. It's reported that infiltration by intratumoural B cells was significantly increased in samples from patients with HPV-positive HNSCC compared with samples from patients with HPV-negative disease, and high densities of CD20 + B cells were associated with favourable outcomes in HNSC (Russell et al., 2013; Li Q. et al., 2020; Pretscher et al., 2009). Anthony R. Cillo et al. discovered that germinal center B cells are present across various stages of progression through germinal center reactions in HPV-positive HNSC, while B cells are found in fewer numbers and non-germinal center states in HPV-negative HNSC (Cillo et al., 2020). These reports are in line with our research results, suggesting that CLDN10 may be one of the key molecules that reshape the tumor immune microenvironment and regulate B-cell homeostasis. However, the tumor microenvironment consists of various components, such as T cells, macrophages, fibroblasts, and other subsets, which collectively promote tumor development. Only by comprehensively examining the tumor microenvironment can personalized treatment plans be provided for HNSC patients.

This study distinctly identifies CLDN10 as a differentially expressed gene in HNSC that strongly correlates with immune B cells. Additionally, immunohistochemical validation was performed on an independent cohort of 112 patients. Nevertheless, our study has certain limitations: firstly, although immunohistochemical analysis confirmed the consistency of CLDN10 with CD20 and CD38 expression in the independent cohort, it did not employ detection methods such as multiple immunofluorescence or flow cytometry that can simultaneously label both phenotypic and functional markers, which limited our precise definition of B cell subpopulations. Second, this study was a correlational study and failed to establish a causal relationship between CLDN10 and B cell function regulation. Third, the tumor microenvironment is a complex ecosystem consisting of various components such as T cells, macrophages, and fibroblasts. This study focused only on B cells and insufficiently explored the potential interactions of other components in this network. Finally, in this study cohort, the proportion of HPV-positive patients was relatively low, resulting in a much smaller sample size for the HPV-positive subgroup compared to the HPV-negative group. This distribution pattern objectively reflects the prevalence of HPV infection in non-oropharyngeal head and neck squamous cell carcinomas in the real world. However, the small sample size of the positive subgroup limits the test efficacy of statistical analysis within this subgroup. Therefore, we did not observe a significant association between CLDN10 and prognosis in the HPV-positive group, which may be partly attributed to the risk of Type II error (false negative) due to insufficient sample size, rather than a complete absence of the biological effect. In the future, it is necessary to expand the HPV-positive cohort or conduct multi-center studies to further verify the prognostic value of CLDN10 in this subgroup.

## 5 Conclusion

In summary, this study systematically described the expression characteristics and clinical significance of CLDN10 in head and neck squamous cell carcinoma for the first time. The main findings are as follows: CLDN10 was significantly downregulated in HNSC tissues, especially in the HPV-negative subgroup. Its low expression was significantly associated with advanced clinical pathological features and poor prognosis, and was an independent prognostic factor for HNSC, with potential diagnostic biomarker value. Bioinformatics enrichment analysis and immunohistochemical verification both indicated that the expression level of CLDN10 was significantly positively correlated with the infiltration abundance of B cells in the tumor microenvironment. This association was particularly stable in HPV-negative HNSC. However, B cell populations have high functional heterogeneity, and this study did not precisely distinguish B cell subgroups.

This study revealed the potential prognostic marker role of CLDN10 in HPV-negative HNSC and the new phenomenon of its association with B cell immunity. It provided preliminary evidence for further exploration of the interaction between tight junction proteins and immune cells in the tumor microenvironment. Whether CLDN10 affects the distribution of B cell subgroups and their anti-tumor/pro-tumor functions by regulating the function of tight junctions still needs to be verified through further functional experiments.

## Data Availability

Publicly available datasets were analyzed in this study. This data can be found here: https://xena.ucsc.edu/.
